# Aqueous autotaxin and TGF-βs are promising diagnostic biomarkers for distinguishing open-angle glaucoma subtypes

**DOI:** 10.1038/s41598-021-81048-3

**Published:** 2021-01-14

**Authors:** Nozomi Igarashi, Megumi Honjo, Ryo Asaoka, Makoto Kurano, Yutaka Yatomi, Koji Igarashi, Kazunori Miyata, Toshikatsu Kaburaki, Makoto Aihara

**Affiliations:** 1 Department of Ophthalmology, Graduate School of Medicine, University of Tokyo School of Medicine, 7-3-1 Hongo Bunkyo-ku, Tokyo, 113-8655 Japan; 2 Department of Ophthalmology, Seirei Hamamatsu General Hospital, Shizuoka, Hamamatsu Japan; 3 Seirei Christopher University, Shizuoka, Hamamatsu Japan; 4 Department of Clinical Laboratory Medicine, Graduate School of Medicine, The University of Tokyo, Tokyo, Japan; 5 Department of Clinical Laboratory, The University of Tokyo Hospital, Tokyo, Japan; 6 Bioscience Division, Reagent and Development Management, TOSOH Corporation, Kanagawa, Japan; 7 Miyata Eye Hospital, Miyazaki, Japan; 8 Department of Ophthalmology, Jichi Medical University Saitama Medical Center, Saitama, Japan

**Keywords:** Diagnostic markers, Eye diseases

## Abstract

The purpose of this study is to examine if aqueous autotaxin (ATX) and TGF-β levels could be used for differentiating glaucoma subtypes. This prospective observational study was performed using aqueous humor samples obtained from 281 consecutive patients. Open angle glaucoma patients were classified into three groups: primary open-angle glaucoma (POAG), secondary open-angle glaucoma (SOAG), and exfoliation glaucoma (XFG). Aqueous levels of ATX and TGF-βs were quantified. The AUC as well as sensitivity and specificity for the classification into normal and glaucoma subtypes using four indicators-ATX, TGF-β1, TGF-β2, and TGF-β3, upon the application of three machine learning methods. ATX, TGF-β1, and TGF-β3 were positively correlated with IOP, and ATX was significantly and negatively correlated with the mean deviation. From least absolute shrinkage and selection operator regression analysis, the AUC values to distinguish each subgroup [normal, POAG, SOAG, and XFG] ranged between 0.675 (POAG vs. normal) and 0.966 (XFG vs. normal), when four variables were used. High AUC values were obtained with ATX for discriminating XFG from normal eyes and with TGF-β3 for discriminating XFG from normal eyes, POAG, or SOAG. Aqueous TGF-β and ATX exhibited high diagnostic performance in detecting glaucoma subtypes, and could be promising biomarkers for glaucoma.

## Introduction

Intraocular pressure (IOP) elevation is hypothesized to be derived from increased aqueous humor (AH) outflow resistance, mainly in the conventional pathway. The conventional pathway consists of the trabecular meshwork (TM) and Schlemm’s canal (SC) tissues. In open-angle glaucoma (OAG), primary open-angle glaucoma (POAG), secondary open-angle glaucoma (SOAG), and exfoliation glaucoma (XFG), an abnormal accumulation of extracellular matrix (ECM) is found along the conventional outflow pathway, leading to the disorganization and degeneration of the TM and SC, as well as IOP elevation^1,2^.

Various liquid mediators including transforming growth factor-beta (TGF-β), endothelin-1, connective tissue growth factor (CTGF), and several other cytokines have been reported to be upregulated in the AH and involved in the increased outflow resistance and IOP elevation. Past reports have shown that aqueous TGF-β2 is significantly upregulated in POAG subjects but downregulated in SOAG subjects^3^. Recently, we reported that aqueous autotaxin (ATX) is present at significantly high levels in SOAG subjects, compared with POAG or normal subjects^4^. ATX is a generating enzyme of lysophosphatidic acid, a major bioactive lipid mediator, and is involved in various physiological processes such as fibrosis and cancer survival^5–8^. Human TM (hTM) cells are reported to express three ATX isoforms (α, β, and γ)^9^. We also found that ATX is associated with a large area under the receiver operating characteristic curve (AUC) for glaucoma diagnosis^4^. In addition, we recently reported that in cytomegalovirus (CMV)-positive AH obtained from Posner–Schlossman syndrome (PSS) patients, one of the major SOAG subtypes, the expression of ATX and TGF-β1 was upregulated, and the levels of ATX and TGF-β1 in AH were correlated significantly with each other^10^. Therefore, we speculate that crosstalk may exist between ATX and TGF-βs, suggesting that concurrent levels of these mediators may be promising diagnostic biomarkers.

To our knowledge, this is the first study to investigate the correlation between ATX and TGF-βs in differentiating glaucoma subtypes. In this study, we evaluated the ability of aqueous ATX and TGF-β levels to differentiate glaucoma subtypes.

## Results

### Comparison of TGF-β and ATX levels in the AH among glaucoma subtypes

A total of 281 eyes of 281 patients, including 88 eyes without any ocular complications (normal), 97 POAG eyes, 48 SOAG eyes, and 48 XFG eyes, were included in the study. The demographic characteristics of the study population are listed in Table 1.

**Table 1:** Demographic characteristics of the study population.

Variables	Normal	POAG	SOAG	XFG	*P* value
Patients (n)	88	97	48	48	
Number of eyes (n)	88	97	48	48	
Sex ratio (male:female)	40:48	52:45	32:16	31:17	NS*
**Age (years)**					
Mean ± SD	72.2 ± 9.7	68.4 ± 11.3	61.0 ± 12.1	75.6 ± 9.9	^†^< 0.005, ^††,††††,†††††,††††††^< 0.0001**
[Range]	25–91	34–87	39–87	39–93	
**IOP (mmHg)**					
Mean ± SD	12.9 ± 2.5	16.3 ± 5.1	23.4 ± 10.6	24.2 ± 9.4	^†,††,†††,††††,†††††^< 0.0001**
[Range]	7–20	8–42	7–52	11–48	

*POAG* primary open angle glaucoma, *SOAG* secondary open angle glaucoma, *XFG* exfoliation glaucoma, *IOP* intraocular pressure.

*Fisher's exact test.

**Steel–Dwass test.

^†^Statistically significant difference between normal and POAG (Steel–Dwass test).

^††^Statistically significant difference between normal and SOAG (Steel–Dwass test).

^†††^Statistically significant difference between normal and XFG (Steel–Dwass test).

^††††^Statistically significant difference between POAG and SOAG (Steel–Dwass test).

^†††††^Statistically significant difference between POAG and XFG (Steel–Dwass test).

^††††††^Statistically significant difference between SOAG and XFG (Steel–Dwass test).

**Figure 1:**
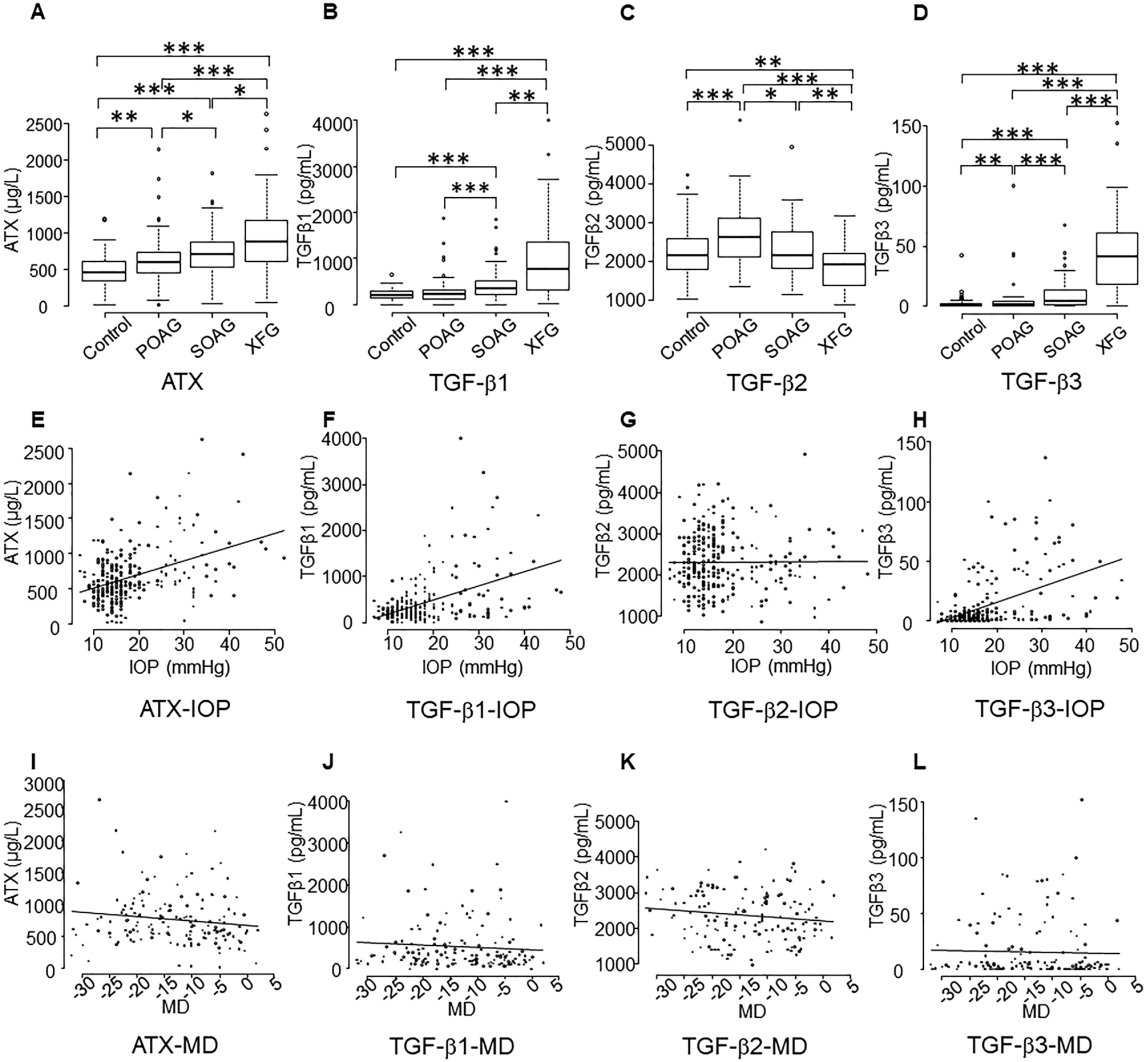
Relationships between aqueous autotaxin (ATX), transforming growth factor-beta 1 (TGF-β1), TGF-β2, and TGF-β3 levels and glaucoma subtypes, intraocular pressure (IOP), and the mean deviation (MD). (**A**–**D**) Relationships between aqueous ATX (**A**), TGF-β1 (**B**), TGF-β2 (**C**) and TGF-β3 (**D**) levels and glaucoma subtypes. (**A**) ATX levels measured via an immunoenzymetric assay were significantly higher in the exfoliation glaucoma (XFG) group compared with the other groups. Also, there was significant differences between the secondary open-angle glaucoma (SOAG) group and the normal (Control) and primary open-angle glaucoma (POAG) groups, and between the Control and POAG groups. (**B**) Aqueous TGF-β1 levels were significantly higher in the XFG group compared with the other groups. Also, TGF-β1 levels in the SOAG group were significantly higher compared with the normal and POAG groups. (**C**) Aqueous TGF-β2 levels were significantly higher in the POAG groups compared to the other groups, and TGF-β2 levels in the XFG group were significantly lower compared to the Control and SOAG groups. (**D**) Aqueous TGF-β3 levels were significantly higher in the XFG group compared to the other groups. TGF-β3 levels in the SOAG group were significantly higher compared with the Control and POAG groups, and significantly higher in the POAG group compared to the Control group. *P < 0.05, **P < 0.01, ***P < 0.001. (**E**–**H**) Correlations between IOP and aqueous ATX (**E**), TGF-β1 (**F**), TGF-β2 (**G**), and TGF-β3 (**H**). ATX (**E**; Spearman’s rank correlation coefficient = 0.315, *P* = 0.0000000663), TGF-β1 (**F**; Spearman’s rank correlation coefficient = 0.336, *P* = 0.00000000964), and TGF-β3 (**H**; Spearman’s rank correlation coefficient = 0.453, *P* = 1.83e−15) were positively correlated with IOP, whereas TGF-β2 (**G**) was not (Spearman’s rank correlation coefficient = 0.0238, *P* = 0.693). (**I**–**L**) Relationships between the MD and aqueous ATX (**I**), TGF-β1 (**J**), TGF-β2 (**K**), and TGF-β3 (**L**). Correlations between the MD and aqueous levels of ATX, TGF-β1, TGF-β2, and TGF-β3 were analyzed. Only ATX exhibited a negative correlation with MD (**I**; Spearman’s rank correlation coefficient = − 0.17, *P* = 0.035); TGF-β1 (**J**; Spearman’s rank correlation coefficient = − 0.155, *P* = 0.0576), TGF-β2 (**K**; Spearman’s rank correlation coefficient = − 0.144, *P* = 0.0788), and TGF-β3 (**L**; Spearman’s rank correlation coefficient = − 0.122, *P* = 0.135) did not exhibit significant correlations.

Aqueous ATX levels were significantly higher in the XFG group compared with the other groups (Fig. 1A; *P* < 0.001 vs. normal and POAG groups, and *P* < 0.05 vs. SOAG group). Also, there were significant differences between SOAG and normal or POAG eyes, between normal and POAG eyes (Fig. 1A). TGF-β1 levels were significantly higher in the XFG group compared with the other groups (Fig. 1B). TGF-β1 levels were significantly higher in the in SOAG group compared to the normal or POAG group (Fig. 1B). TGF-β2 levels were significantly higher in the POAG group compared with the other groups (Fig. 1C), and levels were significantly lower in the XFG group compared with the normal or SOAG group. TGF-β3 levels were significantly higher in the XFG group compared with the other groups (Fig. 1D). Also, TGF-β3 levels in the SOAG group were significantly higher compared with the normal or POAG group, and significantly higher in the POAG group compared to the normal group (Fig. 1D).

### Correlation between TGF-β/ATX levels and IOP or mean deviation (MD)

Next, we evaluated the correlation between IOP and aqueous ATX, TGF-β1, TGF-β2, and TGF-β3 levels. Figure 1E–H shows the correlations between IOP and ATX and IOP and TGF-β1–β3. ATX (Fig. 1E; *P* < 0.00001), TGF-β1 (Fig. 1F; *P* < 0.00001), and TGF-β3 (Fig. 1H; *P* < 0.00001) were positively correlated with IOP, whereas TGF-β2 (Fig. 1G) was not correlated with IOP (*P* = 0.693).

We also evaluated the correlations between ATX and TGF-β1–β3 and the MD (Fig. 1I–L). Only ATX exhibited a negative correlation with the MD (Fig. 1I; *P* = 0.035); TGF-β1–β3 did not exhibit any significant correlations (Fig. 1J–L).

### Receiver operating characteristic curves from LASSO with row values of ATX, TGF-β1, TGF-β2, TGF-β3

**Figure 2:**
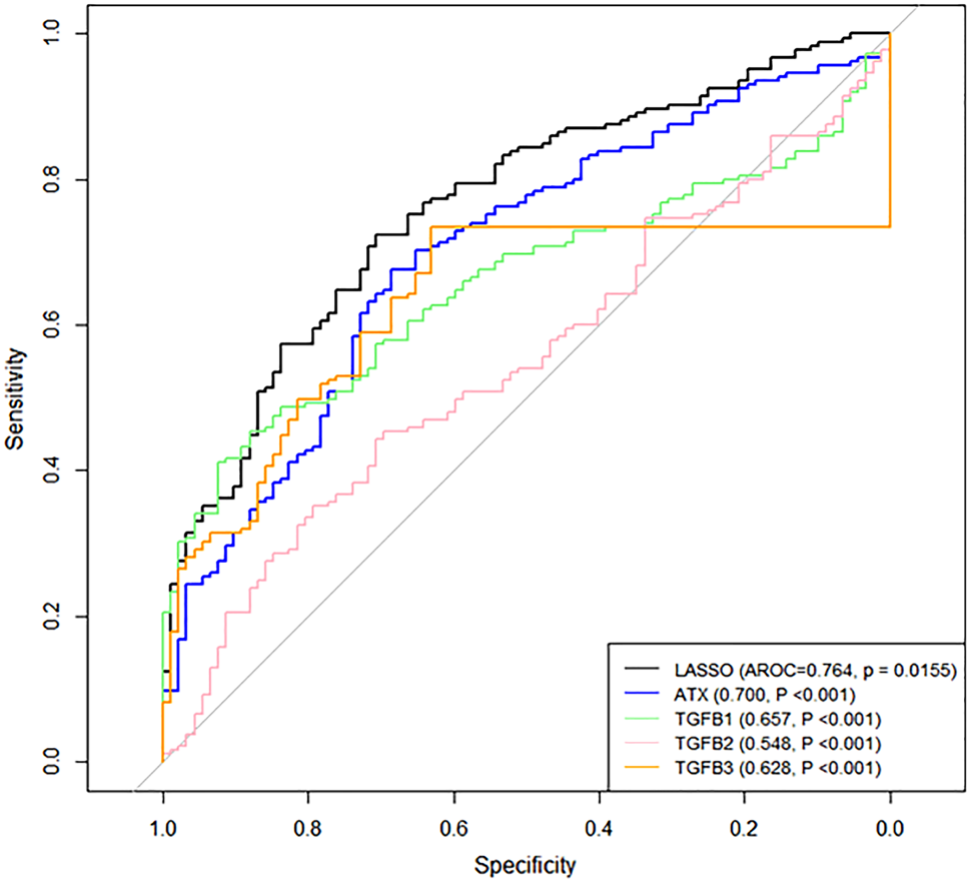
ROC curves with row values for ATX, TGF-β1, TGF-β2, TGF-β3 produced using LASSO regression. The AUC values produced by LASSO were significantly larger compared with the row values of ATX, TGF-β1, TGF-β2, and TGF-β3 (*P* < 0.05). ROC, receiver operating characteristic; LASSO, least absolute shrinkage and selection operator; AUC, area under the receiver operating characteristic curve.

Figure 2 shows the receiver operating characteristic curves representing the row values of ATX, TGF-β1, TGF-β2, and TGF-β3—0.700, 0.657, 0.548, and 0.628, respectively.

Using ATX, TGF-β1, TGF-β2, and TGF-β3, the RF algorithm, SVM and LASSO yielded AUC values of 0.764, 0.762, and 0.713, respectively. The AUC values for ATX, TGF-β1, TGF-β2, and TGF-β3 produced using LASSO differed significantly from those produced with other methods (*P* = 0.0155, < 0.001, < 0.001, and < 0.001, respectively). DeLong’s method with an adjustment based on Holm’s method). Basing on these results, we used LASSO rather than SVM and RF in the subsequent analyses.

Table 2 shows the AUC values for classifying each disease type. The AUC values for distinguishing each subgroup (normal, POAG, SOAG, and XFG) using ATX, TGF-β1, TGF-β2, and TGF-β3 in LASSO regression ranged between 0.675 (POAG vs. normal) and 0.966 (XFG vs. normal). These values did not change significantly when only ATX and TGF-β3 were analyzed using another method (*P* > 0.05, DeLong’s method with an adjustment using Holm’s method). The coefficients of the LASSO regression model after normalizing each of the four variables are shown in Table 3.

**Table 2:** AUC values for classifying each disease type.

Comparison	LASSO (ATX, TGF-β1, TGF-β2, and TGF-β3)	*P* value*	LASSO (ATX and TGF-β3)	*P* value*	*P* value^†^
POAG and normal	0.675	< 0.001	0.607	< 0.001	0.231
SOAG and normal	0.729	< 0.002	0.747	< 0.002	0.768
XFG and normal	0.966	< 0.003	0.967	< 0.003	0.898
POAG and SOAG	0.670	< 0.004	0.694	< 0.004	1.00
POAG and XFG	0.913	< 0.005	0.860	< 0.005	0.768
SOAG and XFG	0.834	< 0.006	0.854	< 0.006	0.196

*P* values with * means the significance of the AUC value. *P* values with ^†^ shows the results of the comparisons of AUC values between LASSO (ATX, TGF-β1, TGF-β2, and TGF-β3) and LASSO (ATX and TGF-β3).

AUC, area under the receiver operating characteristic curve; POAG, primary open-angle glaucoma; SOAG, secondary open-angle glaucoma; XFG: exfoliation glaucoma; LASSO, least absolute shrinkage and selection operator; ATX, autotaxin; TGF-β, transforming growth factor-beta.

**Table 3:** Coefficients of the LASSO regression models after normalizing ATX, TGF-β1, TGF-β2, and TGF-β3 values.

Comparison	ATX	TGF-β1	TGF-β2	TG-Fβ3
POAG and normal	0.324	–	0.648	0.208
SOAG and normal	0.477	0.866	0.206	0.216
XFG and normal	1.260	–	–	3.709
POAG and SOAG	–	0.415	− 0.246	–
POAG and XFG	0.0974	–	− 0.707	1.893
SOAG and XFG	–	–	− 0.107	2.030

POAG: primary open angle glaucoma, SOAG: secondary open angle glaucoma, XFG: Exfoliation glaucoma, LASSO: Least absolute shrinkage and selection operator, ATX: autotaxin, TGF-β: transforming growth factor-beta.

The coefficient values varied according to the variable and classification group; however, high values were obtained with ATX for discriminating XFG from normal eyes, and when using TGF-β3 to discriminate XFG from normal eyes, POAG, and SOAG.

## Discussion

Glaucoma progression depends on the glaucoma subtype. A high IOP is characteristic in some glaucoma subtypes, such as in SOAG and XFG; however, IOP can vary and although it is one of the clinical findings, it does not always give an exact diagnosis for the glaucoma subtype. Therefore, there is an urgent and unmet need for biomarkers that can differentiate glaucoma subtypes and presume disease progression. In the present study, by examining the differentially expressed mediators in AH, we aimed to identify novel diagnostic and severity presuming biomarkers for glaucoma.

Various mediators have been identified as being upregulated in the AH of glaucoma patients and these have been implicated in glaucoma development either by differential expression or potential involvement in the pathogenesis. Levels of several mediators such as TGF-βs, vascular endothelial growth factor, CTGF, and monocyte chemoattractant protein-1 are reportedly higher in the AH of POAG, XFG, and neovascular glaucoma patients^11–15^. However, to date, few studies have investigated the utility of these mediators as glaucoma biomarkers.

Several in vivo and in vitro studies have suggested that these mediators are upregulated and secreted from TM cells, and influence the tissue in conventional pathways through autocrine or paracrine entities via different intracellular signaling pathways, including Rho/ROCK signaling, Wnt, integrins, PKC, BMPs/SMADs, MAP kinases, and others^9,16–24^, which are mostly the downstream signaling targets of TGF-βs^25^. Among them, Rho/ROCK signaling has been reported as one of the major cascades involved in the increase of outflow resistance in the conventional pathway and resultant IOP elevation^25^.

TGF-β2, a strong fibrotic agent, is upregulated in POAG eyes compared with normal eyes, whereas the levels of aqueous TGF-β2 is somewhat downregulated in SOAG eyes^3^. With the hypothesis that some mediators other than TGF-β2 that stimulate Rho/ROCK signaling might exist in SOAG subjects, we previously reported that aqueous ATX levels were significantly upregulated in SOAG eyes compared with normal or POAG eyes, and ATX was positively correlated with IOP^4^. Therefore, we decided to evaluate the possibility that TGF-βs and ATX could be factors in differentiating glaucoma subtypes as well as levels of glaucoma severity.

First, we compared the IOP and aqueous levels of ATX and TGF-β1–β3 across glaucoma subtypes. As shown in Fig. 1A–D, ATX results in the present study are consistent with those of past reports^4,26^ including ours, in which we found that ATX was significantly upregulated in SOAG and XFG subjects, whereas TGF-β2 was significantly higher in POAG subjects but downregulated in SOAG and XFG subjects^3^. ATX (Fig. 1E), TGF-β1 (Fig. 1F), and TGF-β3 (Fig. 1H) were positively correlated with IOP, whereas TGF-β2 (Fig. 1G) was not.

Aqueous TGF-β1 levels were significantly higher in XFG and SOAG subjects (Fig. 1B; *P* < 0.001). Although it has been reported that TGF-β1 is specifically up-regulated in XFG^27–29^, there are few reports that have compared levels of TGF-β1 among different glaucoma subtypes. Schlötzer-Schrehardt et al. reported that TGF-β1 levels were high in XFG and, interestingly, especially high in glaucoma-positive XFG compared with those in exfoliation syndrome (glaucoma-negative)^28^. This suggests the involvement of TGF-β1 in the pathogenesis of increased outflow resistance in XFG, in concordance with the known role of TGF-β1 in stimulating ECM deposition in many fibrotic disorders. However, as for the high levels of TGF-β1 in SOAG, we recently reported that ATX and TGF-β1 were upregulated in the AH in CMV-positive PSS (glaucoma-positive)^10^. In that study, we also found that ATX and TGF-β1 in the AH of PSS patients are significantly correlated. Additionally, our results showed that ATX is significantly related to IOP; however, TGF-β1 is not^10^. In an in vitro study using hTM cells, both ATX and TGF-β1 were significantly upregulated during CMV infection, which mimics SOAG in vitro, whereas TGF-β1 was upregulated following ATX upregulation. Collectively, ATX appears to be predominantly involved in the pathogenesis of increased IOP in PSS, suggesting the possible role of ATX as a biomarker in SOAG.

TGF-β3 levels were also significantly higher in the XFG and SOAG groups compared with other groups (Fig. 1D), the difference between the normal and POAG groups was significant as well. There are only a few reports about the levels of TGF-β3 in glaucoma^30,31^. It has been reported that the concentrations of TGF-β1 and TGF-β3 are high in exfoliation syndrome or XFG and tend to increase in parallel with disease progression; however, these studies focused on the luxation of the lens capsule and did not evaluate glaucoma severity or IOP.

Although TGF-β1 and -β3 were positively correlated to IOP in our study, Pasquale et al.^32^ reported that in the eye in situ, there was no expression of TGF-β1 and -β3 in the TM of eyes without glaucoma, although TGF-β1 and -β3 may play important roles in the regulation of outflow resistance^33,34^. Higher levels of TGF-β1 and -β3 isoforms are most commonly observed in XFG^28,31^, and it has been speculated that high IOP itself may induce the expression of activated TGF-β1^35^. Taken together with the results of our previous study on PSS^10^, TGF-β1 upregulation may reflect IOP elevation, not as the cause but the result of IOP elevation. As for TGF-β3, Vijay et al.^36^ reported that TGF-β3 is involved in ECM deposition by applying 1 ng/mL TGF-β3 to hTM cells for 4 weeks. Notably, the concentrations reported by that study differed considerably from the clinical data obtained in our study (mean aqueous concentration of TGF-β3, 1.9 pg/mL); here, we assumed that TGF-β1 and -β3 were not crucial factors in the initiation of IOP elevation, although they were thought to have at least some effect on IOP.

Contrary to past studies reporting on the relationship between IOP elevation and TGF-β2 in POAG^37,38^, there was no significant correlation between IOP and TGF-β2, even when the analysis was restricted to POAG in this study population. Considering the significant IOP elevation in SOAG and XFG patients in clinical practice, this is not surprising. We speculate that TGF-β2 may not be the major factor that induces IOP elevation when considering the overall glaucoma population, which became one of the important hypotheses we aimed to elucidate in the present study.

To explore the ability to presuming the severity of these mediators, we examined the association between the MD and aqueous levels of ATX or TGF-βs; ATX was the only factor that exhibited a negative correlation to the MD overall. In the present study, various stage of open angle glaucoma patients with wide range of IOP were recruited. We assume that ATX indeed reflected IOP elevation and could be an aqueous biomarker which may predict the possible progression of glaucoma reflecting the IOP elevation. However, glaucoma severity will rather depend on the history of the disease or treatment, further prospective study on analysis between glaucoma progression and ATX and other possible biomarkers with larger subjects will be needed to evaluate the efficacy of biomarkers.

We next evaluated if aqueous ATX and TGF-β1–β3 could be of value in differentiating glaucoma subtypes. We analyzed the AUC values for each aqueous mediator. In a previous study of 164 subjects, in which subjects were divided into normal, POAG, and SOAG groups, ATX produced high AUC values for discriminating glaucoma from normal eyes (0.8871) or SOAG from normal eyes (0.9745)^4^. In the present study, the AUC values for discriminating each subgroup (normal, POAG, SOAG, and XFG) using LASSO regression ranged between 0.675 (POAG vs. normal) and 0.966 (XFG vs. normal) for the four variables used, and these values did not change significantly when only ATX or TGF-β3 was applied. After normalizing each of the four variables, high values were obtained with ATX for discriminating XFG from normal eyes, and with TGF-β3 for discriminating XFG from normal eyes, SOAG and XFG, as well as POAG from XFG (Table 3). Collectively, ATX and TGF-βs can be used as biomarkers to differentiate glaucoma subtypes; specifically, TGF-β3 and ATX have the ability to discriminate XFG from other subtypes or normal eyes. Although XFG is frequently associated with extremely high IOP and presents with a deposit of white fibrillar material on the anterior lens surface or pupillary border as characteristic and diagnostic features, the fibrillar material is not prominent; this feature of XFG is subclinical in some cases, which may lead to misdiagnosis. As XFG is resistant to glaucoma treatment, discriminating XFG is important. If we can measure the levels of ATX and TGF-β1–β3 in the AH, it would provide information in addition to clinical findings to differentiate glaucoma subtypes.

Our study had several limitations. First, we lacked baseline IOP data as most of the glaucoma subjects had already been administered glaucoma eyedrops during their first visit to our outpatient facilities. Further investigations need to include glaucoma patients who had not been administered glaucoma eyedrops. Second, AH samples for SOAG included those from patients with elevated IOP due to various causes; thus, more discrimination would be needed. Third, this is a clinical study; in vitro or in vivo studies are necessary to confirm the mechanism underlying the changes in aqueous ATX and TGF-βs induced in glaucoma.

In the present study, we found that ATX, TGF-β1, TGF-β2, and TGF-β3 can be used as novel diagnostic biomarkers to differentiate glaucoma subtypes. Especially, TGF-β3 and ATX can potentially distinguish XFG, and ATX is effective in presuming severity in glaucoma. Collectively, aqueous TGF-β and ATX levels could be promising biomarkers for glaucoma.

## Methods

### Patients and samples from patients who underwent cataract and glaucoma surgery

AH samples were obtained from cataract or glaucoma patients ≥ 20 years of age who underwent cataract surgery or glaucoma surgery from March 2014 to December 2019 at the University of Tokyo Hospital and three affiliated eye clinics. This prospective observational study was approved by the Institutional Review Board of the University of Tokyo and was registered with the University Hospital Medical Information Network Clinical Trials Registry of Japan (ID: UMIN000027137). All procedures conformed to the tenets of the Declaration of Helsinki. Written informed consent was obtained from each patient. OAG patients were classified into three groups: POAG, SOAG, and XFG, as previously described^4^. Exclusion criteria included patients with other glaucoma types, including primary angle-closure glaucoma and congenital/developmental glaucoma, and patients with a previous history of intraocular surgery other than small-incision cataract surgery without complications. The IOP was determined using Goldmann applanation tonometry, and the maximum preoperative IOP was evaluated within 3 months prior to AH collection. When both eyes of a patient met the inclusion criteria, only the eye treated first was included in the analyses.

### Surgical procedures

The preoperative AH was obtained at the start of the surgery before any incisional procedures, using limbal paracentesis and a syringe and 30-gauge needle. Approximately 70–100 μL was collected in a PROTEOSAVE SS 1.5 mL Slimtube (Sumitomo Bakelite, Tokyo, Japan), registered, and stored at − 80 °C until processing.

### Measurement of ATX, ATX isoforms, and TGF-β1, -β2, and -β3 in the AH

AH samples were collected as described previously^4,10^. Levels of ATX in the AH were determined via a two-site immunoenzymatic assay with an ATX assay reagent using a Tosoh AIA system (Tosoh, Tokyo, Japan). TGF-β levels in the AH were measured using a Bio-Plex Pro TGF-β assay (Bio-Rad Laboratories, Hercules, CA, USA), following the manufacturer’s protocol.

### Statistical analysis for the comparison among glaucoma subtypes

Data were analyzed using the EZR program (Saitama Medical Center, Hidaka, Japan)^39^. The results are expressed as the means ± standard deviations. The *t*-test, chi-square test, or Fisher’s exact test was used to compare between two variables, and the Steel–Dwass test was used for multiple variables. Differences in the data among the groups were analyzed using one-way analysis of variance and Tukey’s post hoc test. A value of *P* < 0.05 was considered to denote statistical significance.

### Statistical analysis for discriminating between normal eyes and glaucoma subtypes

Eyes were classified into normal and glaucoma subtypes using three machine learning methods—random forest (RF), support vector machine (SVM), and least absolute shrinkage and selection operator (LASSO) regression—to analyze the levels of four variables—ATX, TGF-β1, TGF-β2, and TGF-β3.

In the RF method, several decision trees are constructed, and the averaged value from all individual trees is calculated. Each tree is constructed using a different bootstrap sample from the original data. The boosted trees method is a very similar to RF, but when a misclassification of an individual occurs due to a “weak learner” instance, the weight of that individual increases^40^.

In the SVM method, a “hyperplane” that produces the largest separation margin between two classes is constructed. A “soft margin” allows some errors to occur between the separation hyperplane^41^. The SVM method with a Gaussian radial basis function kernel was used in the current study.

LASSO is a shrinkage method for ordinary least squares linear regression. Here, the sum of the absolute values of the regression coefficients is constrained or penalized.

With each of these methods, the classification performance was investigated through leave-one-out (LOO) cross-validation. In LOO cross-validation, one or both eyes of a single subject was used as validation datapoints, and the remaining subjects were used as training datapoints. This procedure was then repeated until each OAG patient and a healthy subject in the original sample were used once as validation datapoints. In other words, for each individual, only the data from all other subjects were used in the prediction. To evaluate the effect of each variable on the classification, LASSO regression analysis was performed after normalizing each of the four variables.

Diagnostic performance was evaluated using the area under the receiver operating characteristic curve (AUC). A comparison of AUCs was carried out using the DeLong method^42^. The Holm method was used to correct *P* values for the problem of multiple testing^43,44^. In addition, AUC values were also calculated using only ATX and TGF-β3 with LASSO regression.

All statistical analyses above were performed using the statistical programming language R (ver. 3.1.3; The R Foundation for Statistical Computing, Vienna, Austria).
